# Platelet-associated oxidative stress and ADAMTS-13 levels are inversely associated with a poor prognosis in septic shock

**DOI:** 10.1186/cc11951

**Published:** 2013-03-19

**Authors:** L Montini, G De Pascale, MA Pennisi, ES Tanzarella, SL Cutuli, A Occhionero, R De Cristofaro, M Antonelli

**Affiliations:** 1 Catholic University School of Medicine, Rome, Italy; 2 Haemostasis and Thrombosis Center, Catholic University School of Medicine, Rome, Italy

## Introduction

Sepsis causes widespread microvascular injury and thrombosis. Some hemostatic factors mediate the mechanisms involved in sepsis-related organ ischemia and failure. Oxidative stress is also increased in sepsis and reactive oxygen species (ROS) favor secretion of von Willebrand factor (vWF) multimers from endothelium and inhibit vWF proteolysis by ADAMTS-13. Moreover, the enzyme indoleamine-2,3-dioxygenase, an important immune regulator, is activated in sepsis and, through generation of kynurenins, promotes antioxidative and anti-infective activities. This study evaluated the relative role of ADAMTS-13, vWF and fibrinogen in the morbidity and mortality of patients with septic shock (SS). The above hemostatic factors were measured together with kynurenine and plasma protein carbonyls, marker of oxidative stress.

## Methods

One group of 12 patients with SS, defined using standard criteria, was enrolled in the ICU of the 'A. Gemelli' Hospital (Rome, Italy). Biochemical, hematologic and hemodynamic parameters were measured on days 1 to 4, 7, 14 and 21. A group of 12 age-matched and gender-matched healthy subjects was used as controls.

## Results

Low ADAMTS-13 activity was observed in SS patients (268 ± 123 ng/ml vs. 760 ± 80 ng/ml in controls). vWF levels (antigen and activity) were increased ~3-fold compared with controls. Likewise, plasma protein carbonyls and kynurenine were globally increased in patients (2.1 ± 1.5 nmol/mg vs. 0.3 ± 02 nmol/mg and 14.4 ± 9.7 μM vs. 2.3 ± 1.3 μM, respectively). Intra-ICU mortality (3 of 15) was strongly and inversely correlated with carbonyl levels (*P *= 0.04) and platelets (*P *= 0.022).

## Conclusion

Hence, we hypothesize that, in the SS setting, platelets contribute to oxidative stress that counteracts the organ failure-associated mortality. Thus, low platelet count, irrespective of bleedings, may favor mortality in SS patients by generating lower ROS amounts.
